# Phase I/II study of docetaxel and cisplatin with concurrent thoracic radiation therapy for locally advanced non-small-cell lung cancer

**DOI:** 10.1038/sj.bjc.6601217

**Published:** 2003-08-26

**Authors:** K Kiura, H Ueoka, Y Segawa, M Tabata, H Kamei, N Takigawa, S Hiraki, Y Watanabe, A Bessho, K Eguchi, N Okimoto, S Harita, M Takemoto, Y Hiraki, M Harada, M Tanimoto

**Affiliations:** 1 Second Department of Internal Medicine, Okayama University Medical School, Okayama, Japan; 2 Department of Radiology, Okayama University Medical School, Okayama, Japan; 3 Department of Internal Medicine, Okayama Red Cross Hospital, Okayama, Japan; 4 Department of Internal Medicine, Sumitomo Besshi General Hospital, Niihama, Japan; 5 Department of Medicine, Kawasaki Hospital, Kawasaki Medical School, Okayama, Japan; 6 Department of Internal Medicine, Chugoku Chuoh Hospital of the Mutual Aid Association of Public School Teachers, Fukuyama, Japan; 7 Department of Internal Medicine, National Shikoku Cancer Center Hospital, Matsuyama, Japan

**Keywords:** combination chemotherapy, concurrent chemoradiation, lung cancer, cisplatin, docetaxel

## Abstract

Recent studies have suggested the superiority of concomitant over sequential administration of chemotherapy and radiotherapy. Docetaxel and cisplatin have demonstrated efficacy in advanced non-small-cell lung cancer (NSCLC). This study evaluated the safety, toxicity, and antitumour activity of docetaxel/cisplatin with concurrent thoracic radiotherapy for patients with locally advanced NSCLC. Patients with locally advanced NSCLC (stage IIIA or IIIB), good performance status, age ⩽75 years, and adequate organ function were eligible. Both docetaxel and cisplatin were given on days 1, 8, 29, and 36. Doses of docetaxel/cisplatin (mg m^−2^) in the phase I study portion were escalated as follows: 20/30, 25/30, 30/30, 30/35, 30/40, 35/40, 40/40, and 45/40. Beginning on day 1 of chemotherapy, thoracic radiotherapy was given at a total dose of 60 Gy with 2 Gy per fraction over 6 weeks. In the phase I portion, the maximum tolerated doses (MTD) among 33 patients were docetaxel 45 mg m^−2^ and cisplatin 40 mg m^−2^. The major dose-limiting toxicity (DLT) was radiation oesophagitis. The recommended doses (RDs) for the phase II study were docetaxel 40 mg m^−2^ and cisplatin 40 mg m^−2^. A total of 42 patients were entered in the phase II portion. Common toxicities were leukopenia, granulocytopenia, anaemia, and radiation oesophagitis, with frequencies of grade ⩾3 toxicities of 71, 60, 24, and 19%, respectively. Toxicity was significant, but manageable according to the dose and schedule modifications. Dose intensities of docetaxel and cisplatin were 86 and 87%, respectively. Radiotherapy was completed without a delay in 67% of 42 patients. The overall response rate was 79% (95% confidence interval (CI), 66–91%). The median survival time was 23.4+ months with an overall survival rate of 76% at 1 year and 54% at 2 years. In conclusion, chemotherapy with cisplatin plus docetaxel given on days 1, 8, 29, and 36 and concurrent thoracic radiotherapy is efficacious and tolerated in patients with locally advanced NSCLC and should be evaluated in a phase III study.

Stage III non-small-cell lung cancer (NSCLC) represents a large patient population, accounting for approximately 30% of all NSCLC patients (van Meerbeeck, 2001). This stage has been intensively investigated for the last 10 years. Several studies and meta-analyses have documented an improvement in patients with stage III disease treated with chemotherapy followed by thoracic radiation therapy, compared with radiation therapy alone (Marino *et al*, 1995; Dillman *et al*, 1996; Sause *et al*, 2000). Full-dose cisplatin-based chemotherapy with concurrent thoracic radiation therapy produced encouraging results with relatively severe toxicities (Lee *et al*, 1996; Reboul *et al*, 1996; Segawa *et al*, 2000). Furthermore, results of a randomised trial demonstrated that concurrent administration of cisplatin-based chemotherapy and radiation significantly improved response rate and median survival compared with sequential administration (Furuse *et al*, 1999); this finding is being confirmed by the Radiation Therapy Oncology Group (RTOG) 9410 trial (Curran *et al*, 2000). Recently, several new agents with potent activity in the treatment of NSCLC have become available. The use of combination chemotherapy including these new drugs has improved the survival of patients with advanced NSCLC (Giaccone *et al*, 1998). The feasibilities obtained with concomitant chemoradiotherapy regimens that include new agents are being reported (Greco *et al*, 1996; Mauer *et al*, 1998).

Cisplatin causes a synergistic effect when given simultaneously with radiation both *ex vivo* and *in vivo* (Alvarez *et al*, 1978; Dewit, 1987). Docetaxel also shows a potential radiosensitising effect both *ex vivo* and *in vivo* (Mason *et al*, 1997; Creane *et al*, 1999). The combination of docetaxel and cisplatin shows additive effects in lung cancer cell lines (Aoe *et al*, 1999), and the antitumour spectrums of cisplatin and docetaxel on various lung cancer cell lines are completely different (Matsushita *et al*, 1999). In clinical trials, the docetaxel/cisplatin combination is one of the most active treatments of advanced NSCLC (Rodriguez *et al*, 2001). Moreover, patients with relapsed NSCLC treated with single-agent docetaxel had prolonged survival, even after receiving cisplatin-based chemotherapy previously (Shepherd *et al*, 2000). Studies demonstrated that a weekly administration schedule of docetaxel resulted in markedly reduced myelosuppression compared with every 3-week administration (Tomiak *et al*, 1994; Hainsworth *et al*, 1998). Since the weekly schedule may improve therapeutic outcome by increasing the dose intensity of docetaxel while reducing bone marrow toxicity from concurrent radiation therapy, the divided schedule of docetaxel on days on 1, 8, 29, and 36 was also considered to have the same advantage.

Based on these concepts, this phase I/II study was conducted to evaluate the safety, toxicity, antitumour activity, and survival effects of chemotherapy consisting of docetaxel plus cisplatin given on days 1, 8, 29, and 36 and concurrent thoracic radiation therapy in patients with locally advanced NSCLC. We planned to administer both cisplatin and docetaxel in as high doses and as early as possible to pursue both local control and eradication of distant micrometastasis.

## PATIENTS AND METHODS

### Eligibility criteria

Patients with histologically or cytologically confirmed NSCLC, unresectable stage IIIA or IIIB disease, were eligible for the study; however, those with T3N1 disease, malignant pleural effusion, pericardial effusion, or pleural dissemination were excluded. Other entry criteria included previously untreated disease, measurable lesion, Eastern Cooperative Oncology Group (ECOG) performance status (PS) (Oken *et al*, 1982) ⩽1, age ⩽75 years, and no history of malignancy within 5 years of study. Before enrolment, each patient had a complete medical history and physical, laboratory, and staging assessments. The laboratory examinations consisted of complete blood cell count (CBC), serum chemistry and tumour marker analyses, 24-h creatinine clearance evaluation, arterial blood gas analysis, urinalysis, electrocardiogram, and pulmonary function tests. Staging work-up included chest plain radiographs, computed tomography (CT) scan of the chest and abdomen (ultrasonography of the abdomen could be substituted), magnetic resonance imaging of the brain, radionuclide bone scan, and bronchofiberscopy. Mediastinoscopy was not included in the staging work-up. A mediastinal lymph node ⩾10 mm along the short axis by CT scan was defined as a metastatic lymph node (N2-3). Patients were required to have a white blood cell (WBC) count ⩾4000 *μ*l^−1^, platelet (PLT) count ⩾100,000 *μ*l^−1^, haemoglobin level ⩾9 g dl^−1^, serum bilirubin level⩽1.5 mg dl^−1^, serum aspartate aminotransferase and alanine aminotransferase levels ⩽2.5 times the upper normal limit, serum creatinine level ⩽1.5 mg dl^−1^, 24-h creatinine clearance level ⩾60 ml min^−1^, and arterial oxygen pressure (PaO_2_)⩾60 mmHg. Patients were excluded if they had markedly diminished vital capacity and/or forced expiratory volume in 1 s, any serious underlying diseases or complications, or were women who were pregnant, breast feeding, or of child-bearing age. Written informed consent was obtained from all patients.

### Response and toxicity evaluations

For the evaluation of response and toxicity, all patients underwent a CBC and serum chemistry analysis two to three times a week, urinalysis, and chest plain radiograph at least weekly during the treatment and at least monthly thereafter; a CT scan of the chest was taken on days 22 and 50 and every 3 months for 2 years; examinations performed at staging work-up were repeated after the completion of treatment. Response was assessed by extramural reviewers using ECOG criteria (Oken *et al*, 1982). The response rate was determined on an ‘intention-to-treat’ basis. Toxicity was assessed and graded using ECOG common toxicity criteria (Oken *et al*, 1982). The grading of acute oesophageal and pulmonary toxicities due to radiation was in accordance with RTOG/European Organization for Research and Treatment of Cancer (EORTC) radiation acute toxicity criteria (Cox *et al*, 1995). Dose limiting toxicity (DLT) was defined as grade ⩾3 haematologic toxicity lasting 3 days or longer, grade ⩾3 radiation oesophagitis, or any nonhaematologic grade 3 or higher toxicities except hair loss and nausea/vomiting.

### Phase I study

The primary end point of the phase I portion of the study was to determine the maximum-tolerated doses (MTDs) and the recommended doses (RDs) of docetaxel and cisplatin for the phase II study when combined with 60 Gy of concurrent thoracic radiation therapy for patients with locally advanced and surgically unresectable NSCLC.

#### Dose escalation scheme

Dose levels of docetaxel and cisplatin are shown in Table 1

**Table 1:** Dose-escalation scheme and principal toxicities in the phase I portion of the trial (*n*=33)

**Dose level**		**1**	**2**	**3**	**4**	**5**	**6**	**7**	**8**
Docetaxel (mg m^−2^)		20	25	30	30	30	35	40	45
Cisplatin (mg m^−2^)		30	30	30	35	40	40	40	40
No. of patients		6	3	3	3	3	3	6	6
No. of DLTs		2	0	0	0	1	0	1	4
Principal toxicity^a^									
Oesophagitis	G3	2 (2)^b^						1 (1)	2 (2)
Liver	G3	1 (1)							
Granulocytopenia	G3		1			1(1)	1	4 (1)	2 (1)
	G4								2 (1)
Anaemia	G3					1		1	
	G4					1		1	
Thrombocytopenia	G3								1 (1)

DLT=dose-limiting toxicity; G3=grade 3; G4=grade 4.

a Toxicity was assessed and graded using ECOG common toxicity criteria (Oken *et al*, 1982), and grading of acute oesophageal toxicity due to radiation was evaluated in accordance with RTOG/ EORTC radiation acute toxicity criteria (Cox *et al*, 1995).

b Number of patients with grade 3 or 4 toxicity (numbers in parentheses indicate number of patients with the DLT).

. At least three patients were entered at each dose level. If a DLT occurred in two of three initial patients at a particular dose level, then three additional patients were treated at the same dose level to define the frequency of that toxicity. If three of three patients or at least four of six patients experienced the DLT, enrolment at this dose level was ceased, the dose level was determined as the MTD, and the preceding dose level was designated as the RD for the phase II study.

#### Treatment schedule and modifications

**Figure 1:**
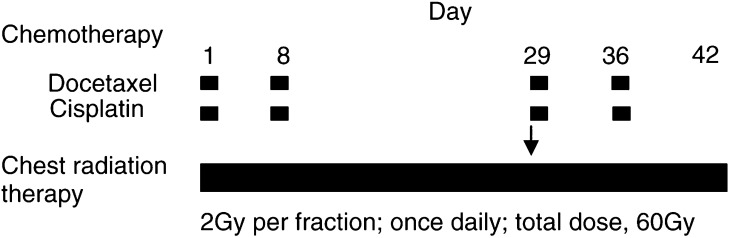
Treatment scheme of the phase I/II study of concurrent chemoradiotherapy with cisplatin and docetaxel in patients with advanced NSCLC. The arrow indicates shrinkage of the radiation field at a total dose of 40 Gy.

The treatment scheme is shown in Figure 1

. Docetaxel was administered intravenously over 1 h followed by 1-h infusion of cisplatin before radiation therapy. Before and after cisplatin instillation, all patients received 2000–2500 ml of normal saline and 5% glucose by infusion over 4 h. Prophylactic antiemetic therapy using 5-hydroxytriptamine type III receptor blocker and dexamethasone was given to all patients. Patients experiencing grade 3 granulocytopenia with infection or grade 4 leukopenia or granulocytopenia subsequently received recombinant human granulocyte colony-stimulating factor (*r*hG-CSF: 2 *μ*g kg^−1^) subcutaneously until leucocyte or neutrophil count recovered to ⩾5000 *μ*l^−1^ or ⩾2000 *μ*l^−1^, respectively. Chemotherapy dose and schedule modifications for toxicity are shown in Table 2

**Table 2:** Chemotherapy and radiotherapy dose and schedule modifications for toxicity^a^

**Toxicity**	**Modification**
*Chemotherapy*	
Grade ⩾3 leukopenia, granulocytopenia, thrombocytopenia, on days 8 or 36	Docetaxel and cisplatin withheld
Grade ⩾3 leukopenia, granulocytopenia, thrombocytopenia, on day 29	Chemotherapy postponed until toxicity recovered to grade 2
Grade 2 leukopenia, granulocytopenia, thrombocytopenia on day 8, 29, or 36	Doses of both drugs reduced by 5 mg m^−2^
24-h creatinine clearance 30–60 ml min^−1^, or serum creatinine 1.5–2.0 mg dl^−1^	Cisplatin dose reduced to 30 mg m^−2^
Creatinine clearance <30 ml min^−1^, or serum creatinine >2.0 mg dl^−1^	Cisplatin withheld
Systemic oedema or fluid retention on day of chemotherapy administration	Docetaxel withheld
Nonhaematologic grade ⩾3 toxicity, except for hair loss or nausea/vomiting	Chemotherapy postponed until recovery
	
*Radiotherapy*	
Grade ⩾3 radiation oesophagitis	Radiation withheld until recovery to grade ⩽2
Lung toxicity (10 Torr↓PaO_2_ from baseline)	Radiation withheld until PaO_2_ decrease is <10 Torr
Grade 3 granulocytopenia with infection or grade 4 leukopenia or granulocytopenia	Radiation discontinued and rhG-CSF (2 *μ*g kg^−1^) administered until leucocyte count ⩾5000 *μ*l^−1^ or neutrophil count ⩾2000 *μ*l^−1^
Grade ⩾3 thrombocytopenia	Radiation withheld until platelet count ⩾25 000 *μ*l^−1^

a Chemotherapy and radiotherapy were discontinued if toxicities did not resolve within 12 weeks.

.

Radiation therapy was administered from day 1 of chemotherapy using a linear accelerator (6–10 MeV), in 2 Gy single daily fractions for five consecutive days each week to a total dose of 60 Gy.

Treatment planning was constructed for a curative radiation field using chest plain radiograph and contrast-enhanced CT scan before concomitant chemoradiotherapy. Principally, the initial radiation field was planned not to exceed 50% of one lung. The initial dose (∼40 Gy) was administered to the original volume that consisted of primary tumour including the movement area by respiration and the ipsilateral hilum with 2 cm margin, and all enlarged mediastinal lymph nodes (⩾10 mm along the short axis) detected by CT scan with 1 cm margin, extending inferiorly to 3 cm below the carina if subcarinal lymph nodes were involved. Other prophylactic radiation fields were not set up. The supraclavicular region was not routinely included if lymph nodes metastasis or primary tumour invasion was not detected. Subsequently, an additional 20 Gy dose was administered to the boost volume, including the sites of primary tumour and hilar/mediatinal lymph nodes according to the tumour and lymph nodes shrinkage determined by contrast-enhanced CT scan on day 29 or later. The original volume was treated with an anterior–posterior parallel-opposed pair of portals, and the boost volume was treated with the same pair or with a pair of oblique fields if the cumulative radiation dose to the spinal cord exceeded 40 Gy. Radiation therapy dose and schedule modifications for toxicity are shown in Table 2.

The patients were carefully treated on an inpatient basis during the concomitant chemoradiotherapy.

### Phase II study

The primary end points of the phase II portion of the study were objective response rate and safety of this combined treatment modality at the RD level. The secondary end point was the 2-year survival rate. The same patient eligibility requirements, treatment schedules, dose and schedule modifications, and response and toxicity criteria as in the phase I portion of the study applied.

### Statistical considerations

The sample size for the phase II study portion was calculated as 36 patients on the assumption that 70% of patients would respond, with a 95% confidence interval (CI) ± 15%. Assuming that 10% of patients would not be evaluable for response, the accrual goal was 42 patients, including those who received chemoradiotherapy at the RD level in the phase I portion of the trial. The survival time was defined as the period from initiation of treatment to death or last follow-up evaluation, and event-free survival was defined as the period from initiation of treatment to PD or death due to causes other than NSCLC. The survival and event-free survival curves were calculated using the Kaplan and Meier method.

## RESULTS

### Patient characteristics

Between June 1997 and December 1999, 69 patients at Okayama University Hospital and 11 affiliated hospitals in Japan were enrolled in this phase I/II study. All patients had ECOG PS of 0–1. In the phase I portion of the study, 33 patients were treated at one of eight dose levels (Table 1). The patients comprised 30 men and three women with a median age of 63 years (range, 29–75 years). Fourteen (42%) patients had squamous cell carcinoma, 13 (39%) adenocarcinoma, three (9%) large-cell carcinoma, and three (9%) unclassified carcinoma. Nine (27%) patients had stage IIIA and 24 (73%) stage IIIB disease. Forty-two patients, including six who had received chemoradiotherapy at the RD level in phase I, were analysed in the phase II portion of the study (Table 3

**Table 3:** Patient characteristics (phase II portion)

	**No. of patients**
No. of patients evaluated/eligible	42/42
Median age, year (range)	67 (29–75)
Sex: male/female	36/6
ECOG PS: 0/1	18/24
Weight loss	
5% or more	5
Less than 5%	37
Histology	
Adenocarcinoma	12
Squamous cell carcinoma	26
Unclassified	4
Stage of disease: IIIA/ IIIB	8/34
TNM classification	
T2N2M0	5
T3N2M0	3
T1N3M0	2
T2N3M0	5
T3N3M0	4
T4N0M0	3
T4N1M0	3
T4N2M0	15
T4N3M0	2

ECOG=Eastern Corporative Oncology Group; PS=performance status.

). Overall, 36 men and six women with a median age of 67 years (range, 29–75 years) were included. Twenty-six (62%) patients had squamous cell carcinoma, 12 (29%) adenocarcinoma, and four (10%) unclassified carcinoma. Eight (19%) patients had stage IIIA and 34 (81%) had stage IIIB disease including three (7%) with swelling of supraclavicular lymph nodes. Five (12%) patients had 5% or more weight loss within 6 months.

### Phase I study

#### Dose escalation and toxicity

The dose-escalation scheme and principal toxicities observed in the phase I portion of the study are summarised in Table 1. At the first dose level, two of the initial three patients developed grade 3 radiation oesophagitis that lasted for 19 and 8 days, respectively, and one of the two patients had grade 3 hepatic toxicity. None of three additional patients treated at the first dose level developed DLT. At dose level 5, one patient encountered grade 3 granulocytopenia on day 72, which continued to day 82. Although six patients were enrolled at dose level 7 for the safety, only one patient experienced DLT (grade 3 radiation oesophagitis and grade 3 granulocytopenia). Four of six patients at dose level 8 developed DLT (two with grade 3 radiation oesophagitis, one each with grade 4 granulocytopenia, and grade 3 granulocytopenia and thrombocytopenia). The MTD was determined to be dose level 8 (docetaxel 45 mg m^−2^, cisplatin 40 mg m^−2^), and dose level 7 (docetaxel 40 mg m^−2^, cisplatin 40 mg m^−2^) was adopted as the RD for the phase II study.

#### Response and survival

**Figure 2:**
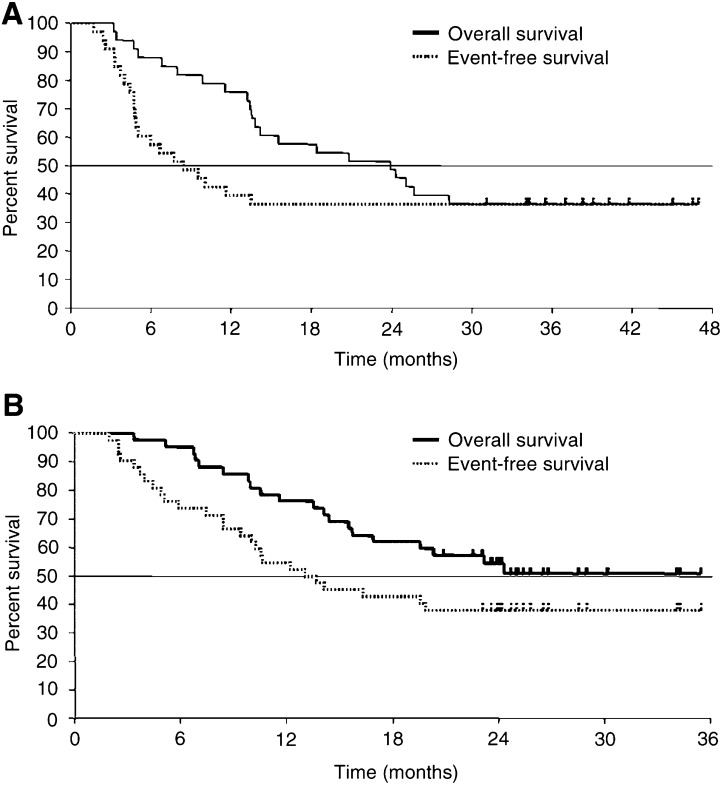
(**A**) Overall (solid line) and event-free (dotted line) survival for 33 patients with locally advanced NSCLC in the phase I portion of the study. (**B**) Overall (solid line) and event-free (dotted line) survival for 42 patients with locally advanced NSCLC in the phase II portion of the study. Censored cases are denoted by tics.

Among 33 entered patients, one (3%) had complete response (CR), 22 (67%) had partial response (PR), eight (24%) had no change (NC), and one (3%) had progressive disease (PD), for an overall response rate of 70% (95% CI, 55–85%). Overall and event-free survival curves for the 33 patients are shown in Figure 2A

. At a median follow-up time of 39 months (range, 32–51 months), 21 (64%) patients had died and 12 (36%) were still alive without disease. The causes of death were directly related to NSCLC in 20 patients and unrelated in one (suicide). Overall survival rates at 1, 2, and 3 years were 76, 49, and 36%, respectively. The median survival time was 24 months. Event-free survival rates (mean) at 1, 2, and 3 years were 39, 36, and 36%, respectively.

### Phase II study

#### Response and survival

Responses in 42 patients entered in the phase II portion of the study were CR in one (2%), PR in 32 (76%), NC in eight (19%), and PD in one (2%), for an overall response rate of 79% (95% CI, 66–91%). Figure 2B shows overall survival for the 42 patients. At a median follow-up time of 26 months (range, 21–36 months), 20 (48%) patients had died and 22 (52%) were still alive. The causes of death were directly related to NSCLC in 19 patients and pneumonia and radiation pneumonitis in one. Overall survival rates (mean) at 1 and 2 years were 76 and 54%, respectively. The median survival time was 23.4+ months. Event-free survival rates at 1 and 2 years were 55 and 38%, respectively.

#### Toxicity

Toxicities observed in 42 patients during treatment and follow-up are listed in Tables 4

**Table 4:** Haematologic toxicities (phase II portion) (*n*=42)

	**No. of patients with grade**					
	**0**	**1**	**2**	**3**	**4**	**% of toxicities grade ⩾3**
Leukopenia	0	2	10	24	6	71
Granulocytopenia	2	3	12	18	7	60
Anaemia	1	12	19	9	1	24
Thrombocytopenia	35	2	2	3	0	7

Haematologic toxicity was assessed and graded using ECOG common toxicity criteria (Oken *et al*, 1982).

and 5

**Table 5:** Nonhaematologic toxicities (phase II portion) (*n*=42)

	**No. of patients with grade**						
	**0**	**1**	**2**	**3**	**4**	**5**	**% of toxicities grade ⩾3**
Nausea/vomiting	9	13	15	5	0	0	11.9
Diarrhoea	28	6	5	2	1	0	7.1
Stomatitis	37	4	0	0	1	0	2.4
Neurologic	40	2	0	0	0	0	0
Liver	38	1	2	1	0	0	2.4
Renal dysfunction	41	1	0	0	0	0	0
Fever	36	1	5	0	0	0	0
Alopecia	2	28	12	—	—	—	—
Oesophagitis	13	12	9	7	1	0	19.0
Pneumonitis	38	3	0	1	0	1	4.8

Nonhaematologic toxicity was assessed and graded using ECOG common toxicity criteria (Oken *et al*, 1982), and grading of acute oesophageal and pulmonary toxicities due to radiation was in accordance with RTOG/EORTC radiation acute toxicity criteria (Cox *et al*, 1995).

. The most common toxicity was leukopenia, which often occurred in conjunction with granulocytopenia. Grade ⩾3 leukopenia and granulocytopenia occurred in 30 (71%) and 25 (60%) patients, respectively. Recombinant human granulocyte colony stimulating factor was administered to 18 (43%) patients for a median duration of 4 days (range, 1–10 days). Grade ⩾3 thrombocytopenia and anaemia occurred in 10 (24%) and three (7%) patients, respectively. Grade ⩾3 radiation oesophagitis, radiation pneumonitis, hepatic dysfunction, and diarrhoea occurred in eight (19%), two (5%), one (2%), and three (7%) patients, respectively. The median WBC count nadir in eight patients with grade ⩾3 radiation oesophagitis was 1150 *μ*l^−1^ (range, 500–2300 *μ*l^−1^); grade ⩾3 leukopenia also occurred in 88% of these eight patients. Overall, 32 (76%) patients experienced any grade ⩾3 toxicity (haematologic only, 45%; nonhaematologic only, 5%; both, 26%). At day 102 from the beginning of radiation therapy, one patient died of pneumonia and radiation pneumonitis. This patient was admitted to a local hospital because of acute respiratory failure, was treated as pneumonia, but died within a week. Autopsy was not carried out.

#### Initial relapse site

Nineteen patients were evaluable for sites of initial relapse in the phase II portion. The primary site was the first site of failure in nine patients (nine without and one with distant metastasis). Distant metastasis was the first site of the failure in 10 patients. Failure sites were as follows: lung (*n*=3), adrenal gland (*n*=2), bone (*n*=2), penis (*n*=1), brain (*n*=1), and skin (*n*=1).

#### Completion of therapy

As shown in Table 6

**Table 6:** Chemotherapy dose intensity

	**Chemotherapy (cycle no.)**					
	**No. 1**	**No. 2**	**No. 3**	**No. 4**	**All**	**Dose intensity^a^**
Cisplatin						87±19%
Real/projected dose (%)	101	99	95	89	96	
No. of patients administered	42	41	40	37	36^b^	
No. of patients administered with dose reduction	0	0	2	0	2	

Docetaxel						86±20%
Real/projected dose (%)	101	99	93	87	95	
No. of patients administered	42	41	40	37	36^b^	
No. of patients administered with dose reduction	0	0	7	6	7	

Median interval between Chemotherapy, days (range)	7	21	7			
	(7–12)	(21–51)	(7–33)			

a Indicates administered dose per time unit/projected dose per time unit (mean±s.d.).

b One patient skipped chemotherapy only on day 8.

, 36 of 42 (86%) patients completed chemotherapy as planned. One patient skipped chemotherapy only on day 8. Doses of cisplatin and docetaxel were reduced in two (5%) and seven (17%) patients, respectively. Reasons for not completing chemotherapy were toxicity (*n*=3), patient refusal (*n*=1), or physician discretion (*n*=2). The median interval (range) between chemotherapy courses 1 and 2, courses 2 and 3, and courses 3 and 4 were 7 days (7–12 days), 21 days (21–51 days), and 7 days (7–33 days), respectively. Ratios of actual to projected doses of cisplatin and docetaxel were 96 and 95%, respectively. As shown in Table 7

**Table 7:** Schedule of chemotherapy compliance

	**Days from the beginning of concomitant chemoradiotherapy**				
	**Cycle No. 1 (on day 1)**	**No. 2 (on day 8)**	**No. 3 (on day 29)**	**No. 4 (on day 36)**	**No. 3 and/or No. 4 (on day 43 or later)**
No. of patients administered (%)	42 (100)	41 (98)	28 (67)	24 (57)	13 (31)

, 28 (67%) and 24 (57%) patients were able to receive chemotherapy on time on days 29 and 36, respectively. On day 43 or later, 13 (31%) patients received cycles 3 and/or 4 of chemotherapy with or without concurrent radiotherapy. Accordingly, the actual dose intensities of cisplatin and docetaxel were 87 and 86%, respectively.

A total of 36 (86%) patients completed radiation therapy (Table 8

**Table 8:** Compliance with radiotherapy

**Thoracic radiation therapy**	**No. (%) of patients**
Completed	36 (86)
without rest period	28 (67)
with rest period	8 (19)
Not completed	6 (14)

); however, eight (19%) required a rest from radiation (median, 10 days; range, 4–28 days) due to granulocytopenia (*n*=6), radiation oesophagitis (*n*=1), and granulocytopenia plus oesophagitis (*n*=1). Reasons for not completing radiation therapy were radiation oesophagitis (*n*=3), radiation pneumonitis (*n*=1), patient refusal (*n*=1), or physician discretion (*n*=1). The total mean radiation doses and durations were 57.2 Gy and 45.6 days, respectively. Overall, 57% of 42 patients completed both chemotherapy and radiation therapy without any modifications according to the protocol.

Three (7%) patients who achieved PR (determined by CT scan) after completing chemoradiotherapy underwent surgery, although this was not part of the study protocol. Postsurgically, two patients with T4N2 disease were downstaged to a pathological CR and pT1N0 disease, and one patient with T2N2 disease still had pT1N2 disease. Microscopic assessment of pT1N0 and pT1N2 tumour samples demonstrated only a few scattered viable tumour cells in necrotic tissue of a primary tumour and/or a mediastinal lymph node. These three patients were still alive without recurrence at the last follow-up evaluation (25, 27, and 36 months, respectively).

## DISCUSSION

This phase I/II study demonstrated encouraging results with concomitant chemoradiotherapy using cisplatin and docetaxel in patients with advanced NSCLC. In the phase I portion of the study, the maximum tolerated chemotherapy doses were determined to be docetaxel 45 mg m^−2^ and cisplatin 40 mg m^−2^. The most common DLTs were radiation oesophagitis and myelosuppression. Recommended doses for the phase II study were docetaxel 40 mg m^−2^ and cisplatin 40 mg m^−2^. In the phase II portion of the study, 79% of patients responded to concomitant chemoradiotherapy with cisplatin and docetaxel, with a median survival time >23 months, and survival rates of 76% at 1 year and 54% at 2 years.

One reason for the favourable results may be the dose intensities of cisplatin and docetaxel achieved in this trial. The doses of docetaxel and cisplatin with concurrent standard radiotherapy (60 Gy in 6 weeks) were escalated more than we expected in the phase I portion. We did not plan dose levels 6–8 at the beginning of this trial, because standard doses without concurrent thoracic radiation therapy in Japan were docetaxel 60 mg m^−2^ and cisplatin 80 mg m^−2^ at a 3-week interval (Kubota *et al*, 2002), which corresponded to docetaxel 30 mg m^−2^ and cisplatin 40 mg m^−2^ on days 1 and 8 at dose level 5. In the phase II portion, the projected dose intensity of both cisplatin and docetaxel in combination with 60 Gy standard thoracic radiation therapy was 27 mg m^−2^ week^−1^, which was comparable to full doses of docetaxel and cisplatin at a 3-week interval (Zalcberg *et al*, 1998). This study demonstrated that the divided schedule could result in similar dose intensities as a weekly schedule. Thus, chemotherapy doses with concomitant chemoradiotherapy in the current trial were similar to or higher than those in the previous phase II trials that did not include concurrent thoracic radiotherapy.

Another advantage of the present regimen is the flexibility of the treatment schedule. To reduce toxicity, we often use the divided schedule on days 1 and 8 as previously reported (Ueoka *et al*, 1998, 1999, 2001; Date *et al*, 2002). We conducted a previous, unsuccessful dose-escalation trial of cisplatin/etoposide chemotherapy in a divided schedule with concurrent thoracic radiation therapy (Segawa *et al*, 2003). In that trial, we strictly fixed the chemotherapy administration on days 29 and 36, and dose and schedule modifications were inhibited on days 29 and 36. Based on that failure, this protocol included the flexible dose and schedule modifications. Accordingly, chemotherapy could be fully administered on days 1 and 8 to all except one patient. On days 29, 36, or later, dosing was individualised according to the prescribed dose and schedule modifications.

Toxicity, although significant, was tolerable according to the dose and schedule modifications of the protocol. In the phase II portion of the study, 76% of 42 patients experienced any grade ⩾3 toxicity, most commonly grade ⩾3 granulocytopenia. However, 57% of 42 patients completed both chemotherapy and radiation therapy without any modifications, and 88% of 42 patients were able to complete the planned chemotherapy and 86% completed radiation therapy according to dose and schedule modifications of the protocol. The dose modifications of chemotherapy based on haematogic toxicity were carried out on days 8, 29, and 36. Leukopenia/granulocytopenia is transient and easily controlled by *r*hG-CSF, and life-threatening infections or treatment-related deaths by acute toxicity were not experienced. Grade ⩾3 radiation oesophagitis occurred in 19% of patients, which was comparable to that occurring with other concomitant chemoradiotherapy regimens (e.g., 20% with cisplatin and etoposide (Albain *et al*, 2002), 26% with cisplatin and paclitaxel (Robert *et al*, 2002), and 46% with carboplatin and paclitaxel (Choy *et al*, 1998)). We have observed a low incidence of grade ⩾3 radiation pneumonitis among patients analysed at a median follow-up time of 26 months in the phase II portion. When paclitaxel/cisplatin was combined with concurrent radiation therapy, 20% of grade ⩾3 late lung toxicity including 8% of grade 5 was reported (Robert *et al*, 2002). Mauer also reported two cases (7%) of grade 5 pulmonary toxicity using docetaxel with concurrent radiotherapy. In our trial, the incidence of grade ⩾3 radiation pneumonitis is low. Although careful follow-up for late radiation pneumonitis is needed, there was no additional grade ⩾3 radiation pneumonitis in April 2003.

Chemotherapy was postponed or skipped on day 29 in 33% of the patients and on day 36 in 43% of patients, although the real/projected doses of cisplatin and docetaxel were 96 and 95%, respectively. Radiation treatment delays were observed in 19% of patients, and 14% did not complete radiation therapy. While treatment compliance is very important, a goal of chemoradiotherapy for locally advanced NSCLS is cure. We increased the chemotherapy doses to the limit, even if that slightly decreased compliance to radiotherapy. In this study, dose and schedule modifications worked very well, and there was no acute treatment-related death.

According to the current phase II trials, concomitant chemoradiotherapy is required to attain median survival time exceeding 17 months, survival rates ⩾65% at 1 year and ⩾35 % at 2 years, and ⩽50% grade 3/4 toxicity in stage III NSCLC (Gandara *et al*, 2001). Survival has to be balanced against toxicity and compliance. The current trial succeeds in achieving satisfactory 1-year and 2-year survival rates, whereas improvements are needed in reducing toxicities and enhancing compliance in the future.

Recently, the Southwest Oncology Group (SWOG) trial 9504 evaluated docetaxel as consolidation therapy after full-dose cisplatin and etoposide with concurrent radiotherapy (Gandara *et al*, 2001). The updated results were very impressive, with a 26-month median survival time and survival rates of 76% at 1 year and 53% at 2 years, although patients with pathological stage IIIB disease were enrolled. Direct comparison of the results is very difficult, because our study included eight (19%) patients with stage IIIA disease in the phase II portion and excluded minimal pathological N2 or N3 disease by mediastinoscopy. The treatment period of our schedule was shorter by at least 9 weeks compared with that in SWOG 9504. For the patients with stage III disease who receive adjuvant surgery, the shorter treatment period might have an advantage.

Many clinical trials are now assessing combinations of taxanes, platinums, and concurrent radiotherapy for patients with locally advanced NSCLC. Frasci *et al* (1997) suggested that weekly cisplatin/paclitaxel could be safely administered with concurrent standard radiotherapy. Robert *et al* (2002) reported the feasibility of cisplatin/paclitaxel and conventional radiation therapy. Wu *et al* (2002) reported that the RDs of docetaxel and cisplatin administered weekly with concurrent radiotherapy were both 20 mg m^−2^. Choy *et al* (1998) reported a phase II study of weekly paclitaxel (50 mg m^−2^) and carboplatin at area under the curve 2 with concurrent radiation therapy.

It is very difficult to select the ideal drugs for concurrent radiotherapy. We prefer to use cisplatin in a concomitant chemoradiotherapy regimen when we pursue the CR. First, Schaake-Koning *et al* (1992) demonstrated that daily cisplatin acted as a radiosensitiser in a phase III trial as well as in *ex vivo* and *in vivo* experiments. Second, although a Hoosier Oncology Group study reported that cisplatin administered every 3 weeks and concurrent radiotherapy did not improve overall survival, the data indicated possible improved long-term survival with concomitant chemoradiotherapy (Blanke *et al*, 1995). Patients treated with the combination therapy had 3- and 5-year survival rates of 9 and 5%, respectively, whereas the rates were 3 and 2% with radiation alone. Recent results from a large, international phase III trial comparing docetaxel/cisplatin, docetaxel/carboplatin, and vinorelbine/cisplatin seem to favour the docetaxel/cisplatin combination even in patients with advanced NSCLC (Rodriguez *et al*, 2001). Moreover, paclitaxel/cisplatin has shown significantly longer median survival when compared to paclitaxel/carboplatin (Rosell *et al*, 2002).

Taxanes are also very attractive drugs for concomitant chemoradiotherapy. Taxanes are known to enhance radiation sensitivity of tumour cells through processes including the following: (1) reoxygenation of hypoxic cells within the tumour, since taxane-killed cells are removed by apoptosis (Milas *et al*, 1995), (2) arrest of cells in both G_2_ and M phases, the most radiation-sensitive phases of the cell cycle (Tishler *et al*, 1992), and (3) mobilisation of T cells and natural killer cells to the tumour (Mason *et al*, 2001). Although the majority of studies including taxanes have assessed paclitaxel, radiosensitisation with docetaxel has also been demonstrated both *ex vivo* and *in vivo*. (Mason *et al*, 1997; Creane *et al*, 1999) Moreover, docetaxel combined with radiation showed a synergistic or additive effect in NSCLC cell lines tested both *ex vivo* and *in vivo* (Nishizaki *et al*, 2001). In a clinical trial of docetaxel used weekly at the MTD, no grade 4 myelosuppression or peripheral neuropathy was observed (Hainsworth *et al*, 1998). The divided schedule might also prove advantageous when combined with cisplatin and concurrent radiotherapy based on nonoverlapping toxicities like a weekly schedule of docetaxel.

There is no evidence of the superiority of concomitant chemoradiotherapy using new drugs (taxanes, vinorelbine)/cisplatin as compared with the older standard, full-dose, cisplatin-based chemotherapy. Our group is now conducting a phase III trial of docetaxel/cisplatin *vs* mitomycin/vindesine/cisplatin with concurrent thoracic radiotherapy.

In conclusion, a regimen comprising cisplatin, docetaxel, and radiation is an exciting approach in the treatment of locally advanced NSCLC. The results of our study showed that chemotherapy consisting of cisplatin and docetaxel given on days 1, 8, 29, and 36 with concurrent radiation therapy might be the effective treatment modality in patients with locally advanced NSCLC.
